# Computational prediction of the osmoregulation network in *Synechococcus sp*. WH8102

**DOI:** 10.1186/1471-2164-11-291

**Published:** 2010-05-10

**Authors:** Xizeng Mao, Victor Olman, Rhona Stuart, Ian T Paulsen, Brian Palenik, Ying Xu

**Affiliations:** 1 Computational Systems Biology Lab, Department of Biochemistry and Molecular Biology and Institute of Bioinformatics, University of Georgia, Athens, GA 30602, USA; 2 College of Computer Science and Technology, Jilin University, Changchun, Jilin, China; 3 The Institute for Genomic Research and the J. Craig Venter Institute, Rockville, MD 20850, USA; 4 Scripps Institution of Oceanography, University of California, San Diego, La Jolla, CA 92093, USA

## Abstract

**Background:**

Osmotic stress is caused by sudden changes in the impermeable solute concentration around a cell, which induces instantaneous water flow in or out of the cell to balance the concentration. Very little is known about the detailed response mechanism to osmotic stress in marine *Synechococcus*, one of the major oxygenic phototrophic cyanobacterial genera that contribute greatly to the global CO_2 _fixation.

**Results:**

We present here a computational study of the osmoregulation network in response to hyperosmotic stress of *Synechococcus sp *strain *WH8102 *using comparative genome analyses and computational prediction. In this study, we identified the key transporters, synthetases, signal sensor proteins and transcriptional regulator proteins, and found experimentally that of these proteins, 15 genes showed significantly changed expression levels under a mild hyperosmotic stress.

**Conclusions:**

From the predicted network model, we have made a number of interesting observations about *WH8102*. Specifically, we found that (i) the organism likely uses glycine betaine as the major osmolyte, and others such as glucosylglycerol, glucosylglycerate, trehalose, sucrose and arginine as the minor osmolytes, making it efficient and adaptable to its changing environment; and (ii) σ^38^, one of the seven types of σ factors, probably serves as a global regulator coordinating the osmoregulation network and the other relevant networks.

## Background

Osmotic stress refers to the stress on a cell induced by sudden changes in impermeable solute concentrations around a cell that affect the equilibrium with the solution inside the cell. When this happens, water molecules will move in and out of cells by diffusion via the lipid bilayer or the aquaporin channels to regain the equilibrium. The induced water flow across the cell membrane will tend to cause changes in the cell volume, specifically in the cytoplasmic volume, and will induce a number of cellular responses to maintain the homeostasis of the cell's water content [1]. When the solute concentration inside a cell is higher than around the cell, i.e., when under *hypoosmotic *stress, water molecules will flow inwards, potentially causing animal cells to swell and increasing the turgor pressure in plant and bacterial cells. Alternatively when the impermeable solute concentration inside a cell is lower, i.e., when under *hyperosmotic *stress, water will flow outwards, hence shocking the cell. In this paper, we focus on the response system to the hyperosmotic stress caused by salt shock knowing that the general knowledge about the hypoosmotic-stress associated response system in prokaryotes is very limited. Throughout this paper osmoregulation refers to osmoregulation in response to the hyperosmotic stress caused by salt shock.

During evolution, all organisms have developed mechanisms to respond to osmotic stresses (or shocks) through tightly regulating a cell's osmolarity so it stays constant, a vitally important condition for cells to survive under changing environments. This regulation process is called *osmoregulation*. Prokaryotes are known to use two basic osmoregulation mechanisms: (i) the *salt-in-cytoplasm *mechanism involves adjusting the salt concentration in the cytoplasm according to the environmental osmolarity, and (ii) the *organic-osmolyte *mechanism involves accumulating uncharged, water-soluble organic compounds to maintain an osmotic equilibrium with the surrounding medium [2]. Previous studies have found that most of the known prokaryotes use the second mechanism [2], that is to use organic osmolytes such as polyhydric alcohols, sugars, free amino acids and their derivatives, and combinations of urea and methylamines [3] to adjust a cell's osmolarity. It has been observed that the least salt-tolerant organisms tend to use disaccharides as the osmolytes, whereas the more halo-tolerant and halophilic species use sugar-polyols and nitrogen-containing solutes [2].

Typically when a cell is under hyperosmotic stress, Na^+ ^and Cl^- ^quickly move into the cell cytoplasm within several seconds; the excessive toxic Na^+ ^are actively exported by Na^+^/H^+ ^antiporters and the nontoxic K^+ ^are also actively transported into the cell from the environment through its K^+ ^antiporters or symporters to maintain the osmolarity needed by the cell in the first hour; and then transport some compatible osmolytes into the cell from the environment or synthesize them in the cell to replace the K^+ ^surplus within the following several hours [4]. To date, osmoregulation has been well studied in *Bacillus subtilis*, *Escherichia coli *and *yeast*, but very little is known about how osmoregulation works in marine cyanobacteria such as *Synechococcus *and *Prochlorococcus. Synechococcus sp*. strain WH8102 is a model organism for organisms that are known to play a key role in global carbon fixation. Since osmolytes represent an important fraction of the fixed carbon, understanding its allocation among different compounds is useful in building a predictive model of these microorganisms.

We have recently carried out a computational study aiming to predict the osmoregulation network in *WH8102 *by extending and applying a computational protocol for biological network prediction that our group previously developed [5]. This prediction capability consists of three key steps for network prediction, namely (i) construction of template network models for related (model) organisms that have substantial experimental data and possibly known information about the target network, (ii) prediction of operons and functional relatedness among genes in the target genome, and (iii) mapping the template network models to the target genome through orthologous gene mapping that is consistent with the predicted operons and gene associations. This computational protocol has been used to predict the phosphorus assimilation network, the carbon fixation network and the nitrogen assimilation regulatory network in *WH8102 *[5-7]. By adapting this protocol to our target model, we have constructed a (partial) osmoregulation network for each of five selected organisms, i.e., *Aphanothece halophytica (A. halo)*, *Corynebacterium glutamicum *ATCC 13032 (*C. glut*), *Escherichia coli K12 *(*E. coli*), *Persephonella marina *EX-H1 (*P. mari*), *Synechocystis sp*. PCC6803 (*PCC6803)*, that have available experimental data related to osmoregulation; made computational prediction of operons as well as of gene functional relatedness in *WH8102*; and then predicted a model for osmoregulation in *WH8102 *through mapping the template models to *WH8102 *in conjunction and validating some of these predictions with experimental data.

Our study has led to a number of new discoveries about osmoregulation in *WH8102*, including identification of key transporters, synthetases, signal sensor proteins and transcription regulator proteins involved in *WH8102 *osmoregulation. Through analyses of the predicted regulatory network, we have gained a number of new insights about *WH8102*: (i) *WH8102 *likely accumulates and uses glycine betaine as the major osmolyte, and glucosylglycerol, glucosylglycerate, trehalose, sucrose and arginine as the minor osmolytes; and (ii) σ^38^, one of the seven types of σ subunits of the RNA polymerase, probably serves as a global regulator in the osmoregulation network in *WH8102*. To the best of our knowledge, this is the first published study on construction of the osmoregulation network using computation methods for cyanobacteria or any prokaryote.

## Results and Discussion

### Template networks

The osmoregulation process in prokaryotes typically consists of two components. First, under hyperosmotic stress, Na^+ ^will be exported out of the cell and K^+ ^will be transported into the cell as a transient response; and second, some compatible osmolytes will be transported from the environment into or synthesized inside the cell to replace the K^+^. The selection of the osmolytes depends on the duration of the osmotic stress and the availability of the substrates and osmolytes in the surroundings. Different species may prefer different osmolytes.

We have collected, through extensive literature search, 63 genes known to be involved in the osmoregulation network in five species, *Aphanothece halophytica *(*A. halo*), *Corynebacterium glutamicum *ATCC 13032 (*C. glut*), *Escherichia coli K12 *(*E. coli*), *Persephonella marina *EX-H1 (*P. mari*), *Synechocystis sp*. PCC6803 (*PCC6803*), for which the osmoregulation has been studied. The following summarizes what is known about each of the osmoregulation networks in the five species (see Table 1 for details):

**Table 1:** Components in the template models

Organism	Gene	Symbol	Function
	AB094497	GsmT	Betaine synthetases with glycine as the
*A. halo*	AB094498	SdmT	substrate
	
	BAB69459	apNhaP	Na^+^/H^+ ^antiporter

	cg1016	BetP	Betaine transporter
	
*C. glut*	cg0864	MtrB	Two-component system that senses osmotic
	cg0862	MtrA	stress

	b0019	NhaA	Na^+ ^antiporter
	b1186	NhaB	
	
	b0020	NhaR	Na^+^/H^+ ^antiporter regulator
	
	b3290	TrkA	Predominant K^+ ^channel
	b1291	TrkE	
	b1363	TrkG	
	b3893	TrkH	
	
	b3747	Kup	K^+ ^channel playing a major role in neutral or slightly alkaline environments
	
	b0698	KdpA	High-affinity K^+ ^channel playing a major role
	b0697	KdpB	under osmotic stress
	b0696	KdpC	
	b4513	KdpF	
	
	b0694	KdpE	Two component system activating Kdp
*E. coli*	b0695	KdpD	expression under osmotic stress
	
	b0311	BetA	Betaine synthetases with proline as the
	b0312	BetB	substrate
	
	b0314	BetT	Proline transporter
	
	b1896	OtsA	Trehalose synthetases with UDPG as the
	b1897	OtsB	substrate
	
	b2677	ProV	Betaine/proline transporter
	b2678	ProW	
	b2679	ProX	
	
	b2938	SpeA	Arginine synthetase
	
	b3404	EnvZ	Two-component system regulating OmpC and
	b3405	OmpR	OmpF under osmotic stress
	
	b0929	OmpF	Porin-encoding genes
	b2215	OmpC	
	
	b4111	ProP	Betaine/proline transporter
	
	b2741	σ^38^	RNA polymerase, Sigma 38 (Sigma S) factor
	
	b1126	PotA	Putrescine/spermidine transporter
	b1125	PotB	
	b1124	PotC	
	b1123	PotD	
	
	b0854	PotF	Putrescine transporter
	b0855	PotG	
	b0856	PotH	
	b0857	PotI	

*P. mari*	ABX75857	GpgS	Glucosylglycerate synthetase
	ABX75858	GpgP	

	sll0689	NhaS3	Na^+^/H^+ ^antiporter
	
	sll0493	KtrA	Predominant K^+ ^transporter playing a major
	slr1509	KtrB	role in K^+ ^uptake under osmotic stress
	slr1508	KtrE	
	
	slr1728	KdpA	High-affinity K^+ ^channel playing a minor role
	slr1729	KdpB	in K^+ ^uptake under osmotic stress
	slr1730	KdpC	
	
*PCC6803*	slr1731	KdpD	Two component system activating Kdp expression under osmotic stress
	
	sll0045	SpsA	Sucrose synthetase
	
	slr1312	SpeA	Arginine synthetase
	slr0662	SpeA	
	
	slr0747	GgtA	Glucosylglycerol/trehalose/sucrose
	slr0529	GgtB	transporter
	slr0530	GgtC	
	slr0531	GgtD	
	
	sll1546	GgpS	Glucosylglycerol synthetases
	slr0746	GgpP	
	
	sll0306	RpoD	RNA polymerase, Sigma 70 (sigma D) factor

• *A. halo *has three genes encoding a Na^+^/H^+ ^antiporter to export Na^+ ^out of the cell [8] and two synthetases known to be involved in the betaine biosynthesis from glycine, compared to the widely used betaine biosynthesis pathway from choline [9].

• *C. glut *has a two-component system known for sensing osmotic stress [10] and a transporter for uptaking betaine [11];

• *E. coli *has 31 genes known to be involved in the osmoregulation process, namely a two-component system for regulating two major porin-encoding genes [12], two Na^+ ^antiporters [4] and three active K^+ ^transport systems whose activation is determined by the environmental condition [3,13,14], and a number of transporters and synthetases for betaine [3], trehalose [15,16], putrescine and spermidine [17], respectively. σ^38^, one of the seven types of σ subunits of RNA polymerase, is a master regulator in a complex regulatory network that governs the expression of many stationary-phase-inducible genes, and was recently proposed as a global regulator in the osmoregulation network [18,19];

• *P. mari *has two genes known for glucosylglycerate synthesis [20];

• *PCC6803 *has 18 genes known to be involved in osmoregulation process, including two K^+ ^uptake systems in which Ktr plays a major role in K^+ ^uptake under osmotic stress [21], a Na^+^/H^+ ^antiporter [22] and a number of transporters and synthetases for glucosylglycerol [23,24], sucrose [25] and arginine [26]; and

We have used these data as the templates and mapped them to *WH8102*, and built the initial target network in *WH8102*.

### The initial osmoregulation model of *WH8102*

By using P-MAP [27] and BLAST [28], we were able to map the genes from the five (partial) template networks outlined above to 28 genes in *WH8102 *(Additional files 1, 2, 3, 4 and 5), providing the components of our initial osmoregulation network model of *WH8102*. P-MAP maps a template network onto a target genome by finding the orthologous genes of the template in the target genome using both sequence similarity information and operon information [27]. When multiple genes from different organisms are mapped to the same gene in *WH8102*, we choose the mapping from the organism with a closer evolutionary relationship. For example, both OtsA (b1896) in *E. coli *and GgpS (sll1566) in PCC6803 are mapped to SYNW1281 in *WH8102*, we have accepted the mapping from PCC6803. Table 2 shows the mapped gene list, along with a numerical score for each (mapped) gene, representing the level of similarity between the two protein domain structures.

**Table 2:** Components in the initial network model

Source Synonym	Source Symbol	Target Synonym	Operon	DA^1 ^Score	Organism
b3404	EnvZ	SYNW0807	SYNW0807-0808	1	*E. coli*
b3405	OmpR	SYNW0808	SYNW0807-0808	0.99	*E. coli*
sll0689	NhaS3	SYNW0157		0.99	*PCC6803*
sll0493	KtrA	SYNW2169	SYNW2165-2170	0.56	*PCC6803*
slr1509	KtrB	SYNW2168	SYNW2165-2170	0.99	*PCC6803*
slr1508	KtrE	SYNW0663	SYNW0663-0667	0.99	*PCC6803*
b2741	σ^38^	SYNW1621		1	*E. coli*
sll0306	RpoD	SYNW0102	SYNW0101-0102	1	*PCC6803*
b0312	BetB	SYNW1956		0.99	*E. coli*
b0314	BetT	SYNW0229	SYNW0229-0233	0.99	*E. coli*
b4111	ProP	SYNW2494	SYNW2494-2495		*E. coli*
b2677	ProV	SYNW1915	SYNW1915-1917	0.56	*E. coli*
b2678	ProW	SYNW1916	SYNW1915-1917	0.99	*E. coli*
b2679	ProX	SYNW1917	SYNW1915-1917	0.99	*E. coli*
AB094497	GsmT	SYNW1914			*PCC7418*
AB094498	SdmT	SYNW1913			*PCC7418*
sll1566	GgpS	SYNW1281	SYNW1279-1286	0.99	*PCC6803*
slr0746	GgpP	SYNW0860			*PCC6803*
slr0747	GgtA	SYNW1285	SYNW1279-1286	0.99	*PCC6803*
slr0530	GgtC	SYNW1283	SYNW1279-1286	0.99	*PCC6803*
slr0531	GgtD	SYNW1284	SYNW1279-1286	0.99	*PCC6803*
ABX75857	GpgS	SYNW2436			*P.marina*
ABX75858	GpgP	SYNW2434			*P.marina*
slr0662	SpeA	SYNW2359		0.99	*PCC6803*
sll0045	Sps	SYNW2520		0.69	*PCC6803*
b0855	PotG	SYNW1544		0.99	*E. coli*
YP_225044	MtrA	SYNW2246			*C. glut*
YP_225045	MtrB	SYNW0551			*C. glut*

1. DA: domain architecture

When mapping the template networks onto *WH8102*, we noticed that only the Na^+^/H^+ ^antiporter in PCC6803 maps to SYNW0157 (CPA2 family Na+/H+ antiporter) in *WH8102*, while Na^+^/H^+ ^antiporters in *E. coli *and *A.halo *do not have any hits. The three K^+ ^uptake systems in the template organisms can only be partially mapped to *WH8102 *(see Template networks); and only KtrBAE (SYNW2168-2169, 0663), is found in *WH8102 *through mapping, while the other two systems, Kdp and Kup, could not be mapped (see Table 2). This mapping result seems to make sense since (i) it has been reported that the Ktr system, not the Kdp system, plays a major role in the K^+ ^uptake under osmotic stress in *PCC6803*; and (ii) the Kup system functions in a low pH environment in *E. coli *[13] while the living environment of *WH8102 *has a pH value 8.1, suggesting that Kup may not be useful for WH8102.

A number of key osmolyte accumulation systems have been found in *WH8102 *through mapping (see Table 2). Multiple transporters and synthetases for the major osmolyte betaine [11] are identified in *WH8102*: BetT (SYNW0229), BetP (SYNW2494) and ProVWX (SYNW1915-1917) are probably used to uptake betaine from the environment when it is available; GsmT (SYNW1914) and SdmT (SYNW1913) are responsible for synthesizing betaine from glycine; and BetB (SYNW1956) are likely used to synthesize betaine from proline. In addition to the major osmolyte, SpsA (SYNW2520) and SpeA (SYNW2359) are two key enzymes in the sucrose synthesis pathway and arginine synthesis pathway respectively; GgtCDA (SYNW1283-1285) are likely used to uptake glucosylglycerol, trehalose and/or sucrose, and GgpSP (SYNW1281, 0860) are for glucosylglycerol biosynthesis; and GpgSP (SYNW2436, 2434) are used to synthesize glucosylglycerate.

The overall initial osmoregulation model can be summarized as follows: (i) under hyperosmotic stress, *WH8102 *first uptakes K^+ ^possibly through the Ktr system, and then accumulates the major osmolyte betaine as well as some minor osmolytes such as glucosylglycerol, glucosylglycerate, trehalose, sucrose and arginine through the flexible osmolyte accumulation systems; and (ii) σ^38 ^(SYNW1621) may serve as a global regulator to coordinate the K^+ ^uptake and the osmolyte accumulation processes. We have noted that some key subunits of an osmoregulation network are missing from the initial model based on template mapping alone, such as GgtB (see Table 2), so additional information is needed to expand and refine the model.

### The expanded osmoregulation model in WH8102

We have expanded the initial network model through "guilt by association" based on co-location (operons), co-regulation and co-evolution information that we can calculate through comparative genomic analyses. The basic idea of such association-based prediction is that if protein A is in the initial model but B is not, we will consider adding B to the model if A and B are related based on the aforementioned "co-" analyses. Additional file 6 lists the expanded model of the osmoregulation network through execution of the following steps.

#### Expansion based on operon prediction

It is well known that genes in the same operon are functionally related, such as enzymes catalyzing subsequent steps in a metabolic pathway, or forming a protein complex [29]. We added 24 new genes into the initial model based on operon predictions for *WH8102 *[30] (see Additional file 6). Specifically, we added SYNW2165-2167 and 2170 since they share the same operon with SYNW2168-2169 (KtrBA), which could be candidates for the missing subunits of the potassium transporter complex Ktr (see Table 2), although additional experimental studies are needed to validate the prediction. SYNW1279-1280, 1282, 1286 are added to the model since they share the same operons with GgpS (SYNW1281) and GgtCDA (SYNW1283-1285), which are the *WH8102 *candidates for GgtB (b0529) in *E. coli*. We noted that this prediction is also supported by phylogenetic profile-based prediction (see Expansion based on phylogenetic profile analysis). SYNW0230-0233 and SYNW2495 are added since they share operons with BetT (SYNW0229) and ProP (SYNW2494), respectively, which are possible candidates for the missing betaine osmolyte transporter subunits. SYNW2435 is added since it shares the same operon with GpgP (SYNW2434), which might be involved in the glucosylglycerate synthesis pathway. SYNW0552 and SYNW2247-2250 are added since they share the same operon, respectively, with SYNW0552 and SYNW2246, which might be used for osmotic signal transduction. SYNW0664-0667 are added since they share the same operon with KtrE (SYNW0663); and SYNW0101 is added since it shares the same operon with RpoD (SYNW0102).

#### Expansion based on predicted protein-protein interactions

**Figure 1:**
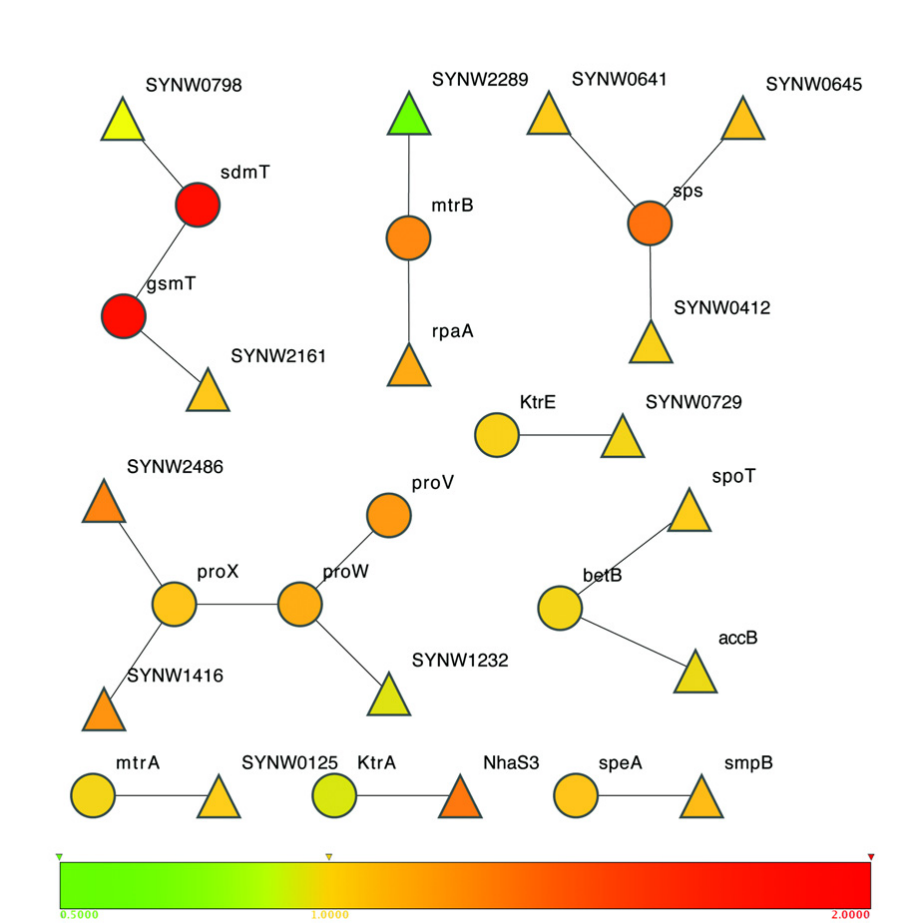
**Genes recruited based on predicted protein-protein interactions**. Circles represent genes in the initial network, and the triangles are the new genes recruited based on protein-protein interactions. The fold-change of gene expression levels in *WH8102 *under hyperosmotic stress against normal conditions is color-coded. (the Cytoscape program [46]).

Protein-protein interactions, derived from large-scale two-hybrid experiments [31] or predicted based on protein fusion analyses [32], provide another source of information for expanding our initial network. We used the set of protein-protein interactions in *WH8102 *predicted previously by our group [5], which contains 950 interactions http://www.cs.uncc.edu/~zcsu/pathways/nitrogen/nitrogen. Specifically, SYNW0798 (a putative transcriptional regulator, ArsR family) and SYNW2141 (possibly a sterol-C-methyltransferase) are added into the network since they are predicted to form a protein complex with SdmT (SYNW1913) and gdmT (1914), respectively, which are already in the initial model. SYNW1232 (possibly a type-3 alternative RNA polymerase sigma factor), SYNW1416 (ABC transporter, nitrate-like) and SYNW2486 (putative cyanate ABC transporter) are added since they are predicted to form a protein complex with ProVWX (SYNW1915-1917). SYNW0412 (hypothetical protein), SYNW0641 (possible glycosyltransferase) and SYNW0645 (putative glycosyltransferase family 2 protein) are added since they are predicted to form a protein complex with Sps (SYNW2520). SYNW2236 (two-component response regulator) and SYNW2289 (two-component response regulator) are added since they are predicted to form a protein complex with SYNW0551. SYNW0125 (putative sugar-binding protein) is added since it is predicted to form a protein complex with SYNW2246. SYNW0034 (biotin carboxyl carrier protein subunit of acetyl-CoA carboxylase) and SYNW2324 (guanosine-3',5'-diphosphate) are added since they are predicted to form a protein complex with BetB (SYNW1956). SYNW0134 (SsrA-binding protein) is added since it is predicted to form a protein complex with SpeA (SYNW2359). SYNW0729 (hypothetical protein) is added since it is predicted to form a protein complex with KtrE (SYNW0663). Overall, 15 new proteins are added into the network based on their predicted interactions with proteins already in the network model (see Figure 1).

#### Expansion based on the σ^38 ^regulon prediction

σ^38 ^has been suggested as a global regulator in the osmotic control of gene expression in *E. coli *[18], so we added additional genes based on orthology mapping of the σ^38 ^regulon of *E. coli *to *WH8102*. We first tested and validated our regulon mapping method in *PCC6803 *(see Materials and Methods) since there are multiple microarray datasets for this organism. We first collected 103 genes of the σ^38 ^regulon in *E. coli *from RegulonDB 6.3 [33], and mapped 49 genes to their orthologs in *PCC6803 *(see Additional file 7). To assess the reliability of our regulon mapping, we collected microarray data of *PCC6803 *under hyperosmotic stress from the public domain (see Additional file 8). We found that 11 of the 49 mapped genes showed more than two-fold change in their expression levels. This result has a significant P-value 0.099 (see Materials and Methods), suggesting that our prediction is statistically significant. The possible reason for the P-value being not any lower could be that (i) the effect of the osmotic stress is rather local; or (ii) the time points at which the expression data are collected do not coincide well with the timing of the relevant response process.

Having demonstrated the reliability of the σ^38 ^mapping on *PCC6803*, we then applied the same mapping to *WH810*, and mapped 41 genes onto *WH8102 *(see Additional file 9). We noticed that some genes in the initial network model, e.g., GgpS (SYNW1281), ProVWX (SYNW1915-1918), BetB (SYNW1956) and ProP (SYNW2494), are predicted as targets of σ^38^, indicating that our mapping functions properly.

#### Expansion based on phylogenetic profile analysis

**Figure 2:**
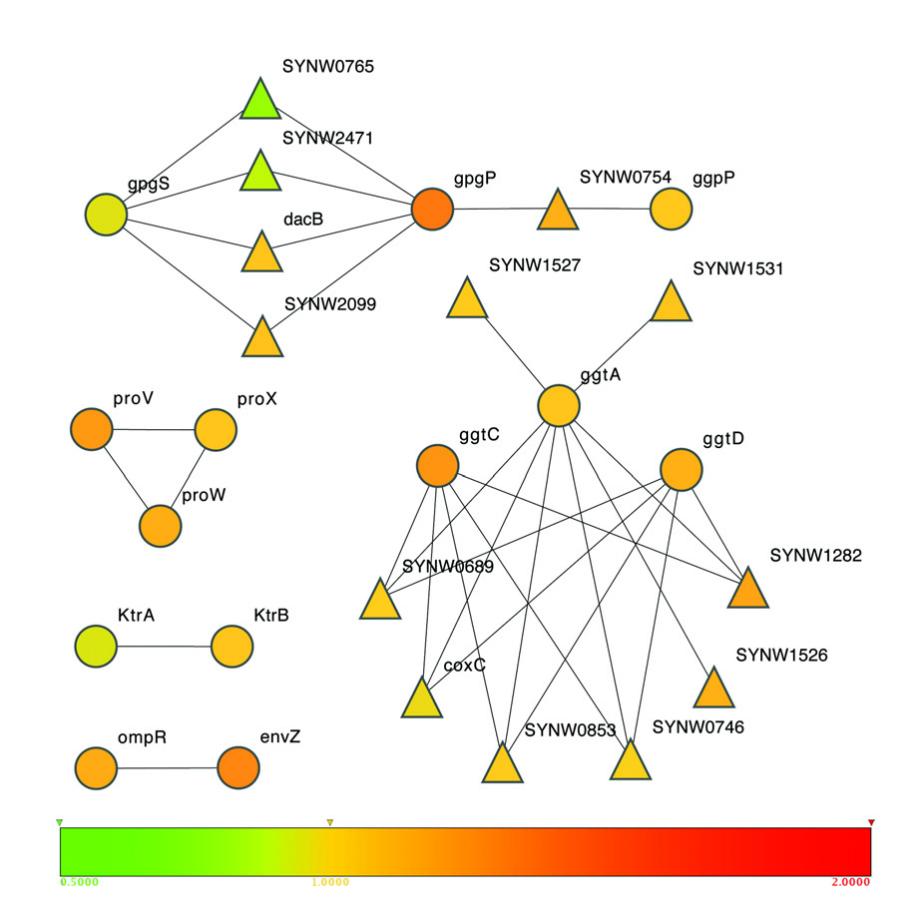
**Genes recruited based on phylogenetic analyses**. Circles represent genes in the initial network model, and the triangles are genes recruited based on phylogenetic analyses. All nodes are color-coded, representing different levels of fold-changes in gene expression in WH8102 under hyperosmotic stress *versus *normal conditions. (the Cytoscape program [46])

It has been well demonstrated that genes with highly similar phylogenetic profiles are functionally related [34]. We used the same 810 bacterial genomes mentioned earlier to construct a phylogenetic profile for each gene in *WH8102 *http://csbl.bmb.uga.edu/~xizeng/research/osmoregulation. Using the phylogenetic profile information, we identified 7 genes already in our initial network and added 13 additional genes to our network model, as shown in Figure 2. Specifically, EnvZ (SYNW0807) and OmpR (SYNW0808), ProVWX (SYNW1916-1918), and KtrBA (SYNW2168-2169) already in our initial network are identified again. SYNW0689, SYNW0746, SYNW0853, SYNW1282, SYNW1526-1527, and SYNW1530-1531 are added since they have very similar phylogenetic profiles with those of GgtCDA (SYNW1283-1285). We believe that they may be candidates for GgtB (b0529) of *E. coli*, and probably involved in glucosylglycerol synthesis. SYNW0754, SYNW0765, SYNW1250, SYNW2099 and SYNW2471 are added since they have very similar phylogenetic profiles with those of GpgSP (SYNW2436, 2434), and they are probably involved in glucosylglycerate synthesis.

Overall, 86 new genes are added to the initial osmoregulation network model, 24 based on co-operon information, 15 based on protein-protein interactions, 41 based on σ^38 ^regulon, and 13 based on predicted co-evolutionary relationships, among which some genes are added by more than one method. For some of these newly added genes, we have predicted their possible functional roles in osmoregulation but for others, all we can say is that we believe that they are involved in osmoregulation in *WH8102 *but we do not have further information about their functional roles. Experimental studies are clearly needed to elucidate the detailed functions of these genes.

### Validation and refinement

To validate the components in the predicted osmoregulation network and refine it based on the validation results, we have checked our predictions against (i) the published literature related to osmoregulation in *WH8102*; (ii) whole-genome microarray gene expression data of both *WH8102 *and *PCC6803*. Our *WH8102 *data is collected under mild salt shock (see Materials and Methods), and the *PCC6803 *data under multiple conditions were collected from public databases; and (iii) protein domain architecture information from public databases.

Our predicted osmoregulation model is highly consistent with the published literature. Lu *et al*. showed experimentally that *WH8102 *synthesizes betaine from glycine using SYNW1913-1914 (SdmT and GsmT) [11], both genes in our initial model. A recent review by Scanlan *et al*. summarized 15 *WH8102 *genes known to be involved in the biosynthesis and uptake of betaine and glucosylglycerol [35], all of which are in our model. Notably, BetAB is considered missing across all cyanobacteria genomes except for *Trichodesmium erythraeum *and *Crocosphaera watsonii*, according to this review. We found a homolog (SYNW1956) in *WH8102 *with an E-value 1.67 × 10^-23^, and included this homolog in our in our model based on the significance of the E-value. Clearly further experiments are needed to study its possible role relevant to osmotic stress.

We have checked our predicted genes against one microarray dataset (Additional file 10) that we collected under mild salt shock. 102 *WH8102 *genes show differential expressions under this condition compared to no salt shock, which is estimated at 1% false discovery rate (see Materials and Methods). Among the 114 genes that we predicted to be involved in the osmoregulation process of *WH8102*, 15 genes show differential expressions under this condition, giving rise to a P-value of 3.44 × 10^-5^, which is the probability of seeing this many genes with differential expressions among 114 randomly selected genes out of the 2,520 *WH8102 *genes. Among these 15 genes, 9 are in the initial model, 4 are added based on operon information, 1 added based on protein-protein interaction data, 3 added based on the regulon prediction and 1 added based on co-evolutionary analyses (see Table 3).

**Table 3:** Validation of the added genes based on different information sources. The P-value is calculated using hyper-geometric distribution.

Methods	Number of recruited genes	Differentially-expressed genes (false discovery rate ≤ 0.01)	P-value
Initial model	27	9	5.24 × 10^-7^
Operon	24	4	1.44 × 10^-2^
Protein-protein interactions	16	1	0.48
Regulon prediction	41	3	0.23
Phylogenetic profile	13	1	0.42
Expanded model	114	15	3.44 × 10^-5^

We also used conservation information of protein domains to validate the gene pairs predicted by P-MAP mapping with one gene from a template genome and the other being the mapped gene in *WH8102*. The consideration is that true orthologous genes across two (related) genomes should in general have the same domain architecture. For each gene in our initial network model as well as genes mapped from the *σ*^38 ^regulon and its orthologous gene from the corresponding reference genome, we calculated a conservation score for their protein-domain architectures, having the score ranging from 0 to 1, with 1 representing two domain architectures being identical and 0 for being totally different (see Materials and Methods). For 63 such gene pairs, 56 pairs have domain-conservation scores almost being 1, indicating that our orthology mapping works properly. seven gene pairs did not have good domain conservation scores, possibly indicating that these gene pairs may not be correctly mapped, which include the following: SYNW2169 (KtrA) does not have PFPF02080, the TrkA C-terminal domain; SYNW1915 (ProV) does not have PF00571, the CBS domain; SYNW2520 (SpsA) does not have PF00862, the sucrose synthase domain; SYNW0944 (idcC) does not have the PF03709, the Orn/Lys/Arg decarboxylase, N-terminal domain; SYNW1884 (SohB) does not have PF08496, the peptidase family S49 N-terminal; SYNW2260 (NhaR) does not have PF03466, the LysR substrate binding domain; and SYNW1759 (TalA) has two additional tandem PF00036, the EF hand domain.

### Crosstalk between osmoregulation and other pathways

**Figure 3:**
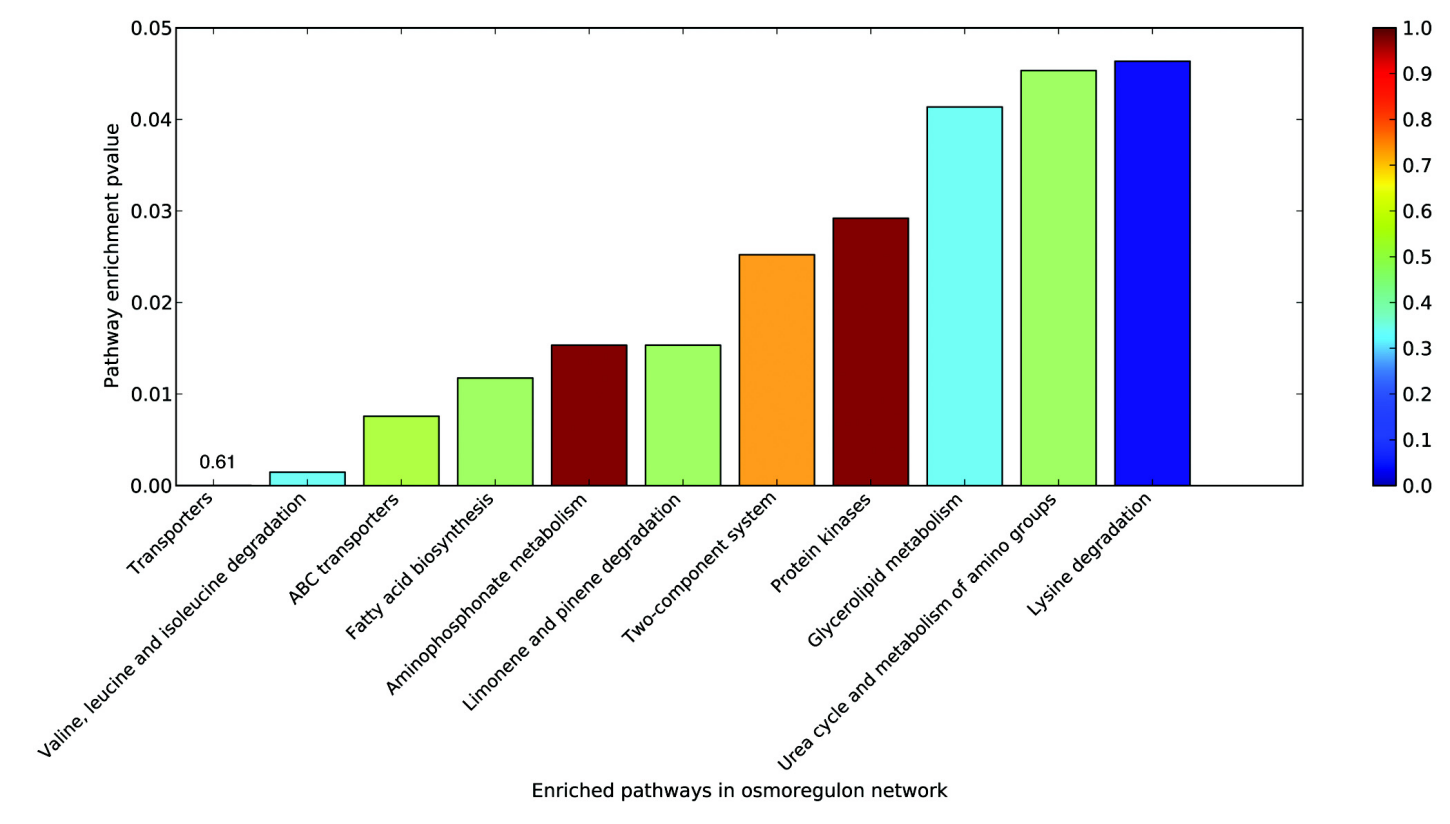
**The enriched pathways within the osmoregulation model of *WH8102***. The proportion of the up-regulated genes against all the genes in the pathway under consideration is color-coded.

We have carried out a pathway-enrichment analysis on our osmoregulation model using KOBAS [36,37] in conjunction with an application of the expression data of *WH8102 *(see Materials and Methods). 87 out of the 114 genes in our model are mapped onto 82 KEGG pathways (see Additional file 11), and 11 pathways are enriched with P-value ≤ 0.05 (see Figure 3), including transporters, valine, leucine and isoleucine degradation, ABC transporters, fatty acid biosynthesis, aminophosphonate metabolism, limonene and pinene degradation, two-component system, protein kinases, glycerolipid metabolism, urea cycle and metabolism of amino groups, lysine degradation. These enrichment results suggest that these pathways may have direct cross-talk with the osmoregulation process.

### A working model for osmoregulation network

**Figure 4:**
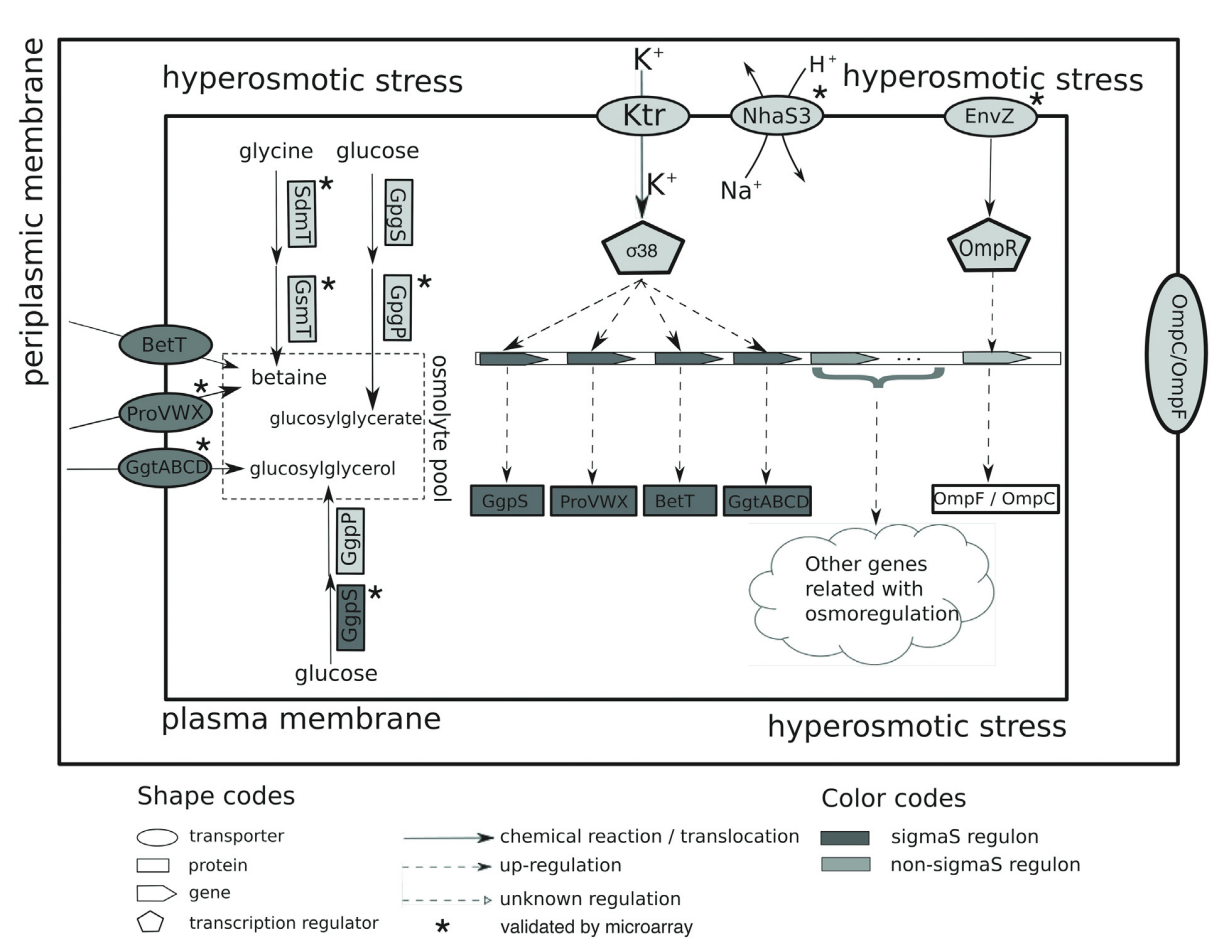
**A working model for the osmoregulation network of *WH8102***. When under hyperosmotic stress, N^+ ^is actively exported by the NhaS3 antiporter; K^+ ^is actively transported into the cell by the Ktr systems, and stabilizes the global transcription regulator σ^38^; this then activates other genes in relation with osmotic response, all represented as deep grey ellipses, including osmolyte transporters or synthetases, such as BetT, ProVWX, GgtABCD and GgpS; hyperosmotic stress also activates the two component system of EnvZ and OmpR to regulate porin protein OmpC and OmpF located in the periplasmic membrane; Besides sigma factor-regulated genes, SdmT and GsmT synthesize the major osmolyte betaine, and GpgS and GpgP to synthesize glucosylglycerol.

We now describe a working model for the osmoregulation network in *WH8102 *based on our prediction and validation results (see Figure 4). Our model consists of 114 genes, 94 of which have predicted functional roles in osmoregulation and the remaining 20 are predicted to be involved in the network but without predicted functions. Our overall model can be summarized as follows, which consists of the following key components and gene assignments.

1. *Na^+ ^export system*: SYNW0157 functions as a Na^+^/H^+ ^antiporter to export the excessive Na^+ ^out of the cell under hyperosmotic stresses;

2. *K^+ ^uptake systems*: SYNW0663, SYNW2168-2169 consist of a Ktr system and may play a major role in active K^+ ^uptake under hyperosmotic stresses;

3. *Osmolyte accumulation systems*: SYNW0229 (BetT), SYNW1915-1917 (ProVWX) and SYNW2494 (ProP) are used to uptake the major osmolyte betaine if available in the environment, while SYNW1913-1914 (SdmT and GsmT) are used to synthesize betaine from glycine when needed; SYNW2436 (GpgS) and SYNW2434 (GpgP) are used to synthesize glucosyl glycerate or mannosylglycerate from glucose; SYNW1281 (GgpS) and SYNW0860 (GgpP) are used to synthesize glucosylglycerol from glucose, while SYNW1283-1287 (GgtABCD) uptake glucosylglycerol when available in the environment; SYNW2359 (SpeA) is for synthesizing the minor osmolyte arginine; and SYNW2520 (Sps) for synthesizing the minor osmolyte sucrose;

4. *Global regulator*: SYNW1621 (σ^38^) functions as a global transcription regulator possibly to bridge the K^+ ^uptake and osmolyte accumulation processes and to coordinate osmoregulation with other biological processes; and

5. *Two-component signal transduction systems*: SYNW0807-0808 (EnvZ and OmpR) regulate water across the outer membrane and gene responses of some osmoregulatory elements; and SYNW0551 and SYNW2246 may be responsible for sensing external osmotic stress and/or activating a number of genes relevant to osmoregulation, which suggests other member genes should also be relevant to osmosensing but further experiments are needed to derive more information about this.

## Conclusions

From our predicted model, we found that (i) *WH8102 *likely accumulates and uses glycine betaine as the major osmolyte, and glucosylglycerol, glucosylglycerate, trehalose, sucrose and arginine as the minor osmolytes; and (ii) σ^38^, one of the seven types of σ subunits of the RNA polymerase, probably serves as a global regulator in the osmoregulation network in *WH8102*. We believe that this working model provides useful information to experimental biologists in their research design for further studying the osmoregulation process in *WH8102*. To the best of our knowledge, this model represents the first published study on construction of the osmoregulation network using computation methods for cyanobacteria or any prokaryote.

## Methods

### Data

All the used genome sequences and annotations were retrieved from NCBI FTP (ftp://ftp.ncbi.nih.gov/genomes/Bacteria, 05-02-2008). The σ^38 ^regulon of *E. coli *are downloaded from RegulonDB 6.3 [33].

### Mapping of template networks

We used the P-MAP program [27] with *E-value *≤ 10^-6^, in conjunction with BLAST [28] with *E-value *≤ 10^-20^, to map template networks to *WH8102*. The mapping results from different organisms were then merged, which gives the initial network model. When different functional roles are assigned to the same gene or one functional role is assigned to different genes based on different templates, we resolved the conflict by assessing the information of evolutionary distance of organism, the conservation of protein domain architectures, and available gene expression values (see below).

### Expansion of initial network

We downloaded the predicted operons of *WH8102 *from the DOOR database [30] and the protein-protein interactions from http://www.cs.uncc.edu/~zcsu/pathways/nitrogen/nitrogen.

**Figure 5:**
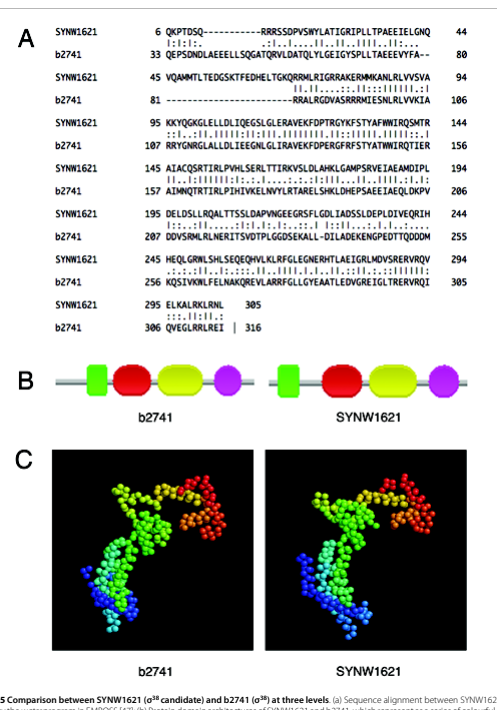
**Comparison between SYNW1621 (σ^38 ^candidate) and b2741 (σ^38^) at three levels**. (a) Sequence alignment between SYNW1621 and b2741 by the *water *program in EMBOSS [47]; (b) Protein domain architectures of SYNW1621 and b2741, which represent as a series of colourful shapes, respectively; (c) the protein 3D structures of SYNW1621 and b2741 predicted using the LOMETS software [48] and Rasmol [49].

We have predicted the σ^38 ^regulon in *WH8102 *based on the following observation: σ^38 ^candidate (SYNW1621) in *WH8102 *has high sequence similarity as well as domain architecture and 3D protein structure similarities with its counterpart b2741 (σ^38^) in *E. coli *(see Figure 5). So we assume that the two regulons, the one in *E. coli *and the one in *WH8102*, have similar sets of components, and hence we have mapped the σ^38 ^regulon of *E. coli *to *WH8102*.

The functional relatedness among *WH8102 *genes is assessed based on the similarities of their phylogenetic profiles calculated against *n *= 810 bacterial genomes. A *phylogenetic profile *of a *WH8102 *gene in reference to the *n *genomes [*G_1_*,..., *G_n_*] is a binary string *a_1_, ..., a_n _*with *a_i _*= 1 if the gene has a detectable ortholog in *G_i _*and *a*_i _= 0 otherwise [34]. Let *m_j _*be the number of *WH8102 *genes having an ortholog in the *j*^*th *^reference genome. Traditionally, the functional relatedness between two genes has been estimated using the Euclidean distance between the phylogenetic profiles of the two genes. Our initial analysis indicates that this is not a very effective way for measuring the functional relatedness of genes so we have modified it as follows. We define the *likelihood *of the *i*^*th *^gene of *WH8102*, *LH_i_*, having orthologs across all the reference genomes as , with *p_j _*= *m_j_/n *if the gene has an ortholog in the *j*^*th *^reference genome and *p_j _*= 1 − *m_j_/n *otherwise. The *functional relatedness *between genes, *g*_i _and *g*_j_, is defined as

with , *d_H _*is the Hamming distance between the phylogenetic profiles of *g*_i _and *g*_j_, α is a weighting factor (the default is α = 2), and *h *is the number of genomes that have orthologs of both *g*_i _and *g*_j_.

Using this measure, we can define for each *WH8102 *gene its functionally most related genes. For each gene *g *in our initial model, we recruit all genes into the model, which are more closely related to *g *than any gene already in the initial model.

### Genome-wide search for domain architecture similarity

We used the Pfam database [38] as the source of protein domain definitions, and the InterproScan program [39] to identify all protein domains in the template genomes and the target genome. To measure the similarity between two given domain architectures, we implemented a domain-architecture similarity score, which was originally defined by Lin *et al *[40] as a linear combination of three different indices: the Jaccard index, which measures the number of common domains that the two architectures contain; the Goodman-Kruska γ function, which estimates the similarity of the arrangement of the distinct domains shared by the two architectures; and the domain duplicate index, which assess the similarity among the duplicated domains in the two architectures. The related scripts can be downloaded from http://csbl.bmb.uga.edu/~xizeng/research/osmoregulation.

### Prediction of differentially expressed genes

We implemented a script based on BioRuby http://bioruby.org to calculate the average fold-change of expression levels for each gene under two conditions if microarray data is available, otherwise we have collected genes from published papers, which were found to be differentially expressed (see Additional file 12). We have used fold-change = 2.0 and 0.5 as the cutoffs for calling a gene up- or down-regulated, respectively, and no change for values in-between.

### DNA microarray transcriptional profiling for WH8102

Microarray data was obtained using a whole-genome microarray, design of which was described previously [41]. Cultures of *Synechococcus *sp. strain *WH8102 *were grown in synthetic ocean water-based media with supplemented nutrients as described previously [6]. This medium is 75% seawater salinity. Triplicate cultures were grown to mid-exponential at which point RNA was extracted from half of each culture and designated as control RNA; and 0.125 M NaCl was added to the other half of each culture bringing its salinity up to approximately that of seawater. After two hours RNA was extracted from the remaining half of the culture. RNA was harvested using a Trizol-based method and purified with a Qiagen RNeasy kit according to the manufacturer's specifications. Microarray hybridization was performed as described previously [6]. Briefly, an indirect labeling method was used to label cDNA with Cy3 (control samples) or Cy5 (treated samples), samples were then pooled and hybridized to the same array. This was done in duplicate for each biological replicate. Additionally, reverse labelling was performed for each biological replicate, resulting in three total technical replicates per biological replicate. Following hybridization, slides were promptly scanned at a 10-μm resolution using an Axon 4000B scanner with GenePix 4.0 software. Processing of the TIFF images from hybridized arrays was performed using TIGR-Spotfinder http://www.tigr.org/software, and the datasets normalized by applying the LOWESS algorithm, using block mode and a smooth parameter of 0.33, available in the TIGR-MIDAS package http://www.tigr.org/software. Statistical analysis was performed on the mean of log2-transformed signal ratios of the replicate spots using the Statistical Analysis of Microarrays (SAM) algorithms [42] with a false discovery rate (FDR) of less than 1%.

### P-value calculation based on microarray data

We use the following hyper-geometric distribution to estimate the cumulative probability that our predictions may happen by randomly drawing genes from all the differentially expressed genes:

where *N *is the number of all genes in the genome, and *A *is the number of differentially expressed genes under hyperosmotic stress; *K *is the number of genes in our network; and *V *is the number of predicted genes in our network that are differentially expressed under hyperosmotic stress.

### Pathway enrichment analysis

Pathway enrichment analysis can usually give biological insights about the genes showing differential expressions under two conditions [43,44]. We used the KOBAS program [36] to identify significantly enriched pathways by genes in our predicted osmoregulation network against all known genes in *WH8102 *as the background. KOBAS maps interested genes onto known KEGG pathway [45] using BLAST with E-value ≤ 1e-5 and rank ≤ 10, and then uses hyper-geometric distribution to calculate the statistical significance of each pathway populated by the interested genes with respect to all the genes encoded in the whole genome as the background. The relevant formulas have been well described in previous work [36].

## Authors' contributions

XM carried out all the computational studies, and drafted the manuscript; VO designed and implemented a scoring function, and participated in phylogenetic analysis; RS, ITP and BP carried out microarray experiment and data analysis, and helped writing the manuscript; YX conceived the study, participated in its design and coordination, and drafted the manuscript. All authors read and approved the final manuscript.

## Supplementary Material

Additional file 1 **Gene Mapping from *A. halo *to *WH8102***. This file contains genes mapped from the template organism *A. halo *to *WH8102 *in the initial osmoregulation network. The file has nine columns: basic gene information including synonym, GI number and symbol in the first-fifth columns, operon information if applicable in the sixth column, and comparison of protein domain architecture if applicable in the seventh-ninth columns. The score in the seven column measures the similarity of protein domain architectures: 1 and 0 means completely identical and different, respectively, and values between them means partially identical. Click here for file

Additional file 2 **Gene Mapping from *C.glut *to *WH8102***. This file contains genes mapped from the template organism *C.glut *to *WH8102 *in the initial osmoregulation network. The file has nine columns: basic gene information including synonym, GI number and symbol in the first-fifth columns, operon information if applicable in the sixth column, and comparison of protein domain architecture if applicable in the seventh-ninth columns. The score in the seven column measures the similarity of protein domain architectures: 1 and 0 means completely identical and different, respectively, and values between them means partially identical. Click here for file

Additional file 3 **Gene Mapping from *E.coli *to *WH8102***. This file contains genes mapped from the template organism *E.coli *to *WH8102 *in the initial osmoregulation network. The file has nine columns: basic gene information including synonym, GI number and symbol in the first-fifth columns, operon information if applicable in the sixth column, and comparison of protein domain architecture if applicable in the seventh-ninth columns. The score in the seven column measures the similarity of protein domain architectures: 1 and 0 means completely identical and different, respectively, and values between them means partially identical. Click here for file

Additional file 4 **Gene Mapping from *P.mari *to *WH8102***. This file contains genes mapped from the template organism *P.mari *to *WH8102 *in the initial osmoregulation network. The file has nine columns: basic gene information including synonym, GI number and symbol in the first-fifth columns, operon information if applicable in the sixth column, and comparison of protein domain architecture if applicable in the seventh-ninth columns. The score in the seven column measures the similarity of protein domain architectures: 1 and 0 means completely identical and different, respectively, and values between them means partially identical. Click here for file

Additional file 5 **Gene Mapping from *PCC6803 *to *WH8102***. This file contains genes mapped from the template organism *PCC6803 *to *WH8102 *in the initial osmoregulation network. The file has nine columns: basic gene information including synonym, GI number and symbol in the first-fifth columns, operon information if applicable in the sixth column, and comparison of protein domain architecture if applicable in the seventh-ninth columns. The score in the seven column measures the similarity of protein domain architectures: 1 and 0 means completely identical and different, respectively, and values between them means partially identical. Click here for file

Additional file 6 **Expanded Model of Osmoregulation in *WH8102***. This file contains an expanded model of osmoregulation network in *WH8102*, including basic gene information (gene synonym, symbol, GI number and annotation) in the first-second and tenth-eleventh columns, computational evidences about cross-organism mapping in the third column and network expanding by guilt-by-association with co-operon, co-interaction, co-regulon and co-evolution information in the four-seven columns, and microarray experimental evidences in the eighth-ninth columns. "X" in the third column means the gene is mapped from a template organism, and "X" in the seven column means that the gene is recruited by sigma 38 regulon. Click here for file

Additional file 7 **Sigma 38 Dependent Genes Mapping from *E. coli *to *PCC6803***. This file contains genes in sigma 38 regulon mapped from the template organism *E. coli *to *PCC6803*. The file has thirteen columns: basic gene information including synonym, GI number and symbol in the first-sixth columns, operon information if applicable in the sixth column, comparison information of protein domain architecture if applicable in the seventh-ninth columns, and microarray experimental data in the tenth-eleventh columns. The score in the seven column measures the similarity of protein domain architectures: 1 and 0 means completely identical and different, respectively, and values between them means partially identical. Click here for file

Additional file 8 **Microarray data of *PCC6803 *collected from the public domain**. This file contains summary of microarray data of *PCC6803 *collected from the public domain including Pubmed ID, experimental condition, gene number and microarray platform. Click here for file

Additional file 9 **Sigma 38 Dependent Genes Mapping from *E. coli *to *WH8102***. This file contains genes in sigma 38 regulon mapped from the template organism *E. coli *to *WH8102*. The file has nine columns: basic gene information including synonym, GI number and symbol in the first-sixth columns, operon information if applicable in the sixth column, comparison information of protein domain architecture if applicable in the seventh-ninth columns. The score in the seven column measures the similarity of protein domain architectures: 1 and 0 means completely identical and different, respectively, and values between them means partially identical. Click here for file

Additional file 10 ***WH8102 *Saltshock Genes With 1%FDR**. This file contains genes with less than 1% false discovery rate under slat shock in *WH8102*, including basic gene information in the first column, statistical significance information in the second-forth columns, and gene expression values under different experimental treats in the fifth-thirteen columns Click here for file

Additional file 11 **Pathway Enrichment Analysis of Osmoregulation Network in WH8102**. This file contains enriched KEGG pathways involved in the osmoregulation network. The P-value in the second column measures the statistical significance of each pathway populated by the genes in the osmoregulation network with respect to all the genes encoded in the whole genome as the background. Click here for file

Additional file 12 **PCC6803 Genome-Wide Gene Expression Under Osmotic Stress**. This file contains expression data of genes in PCC6803 under hyperosmotic stress, collected from published papers. Fold change > 2 means the gene is up-regulated by at least 2 fold, and fold change < 0.5 means the gene is down-regulated by at least 2 fold. Click here for file
